# ORGAVADS: establishment of tumor organoids from head and neck squamous cell carcinoma to assess their response to innovative therapies

**DOI:** 10.1186/s12885-023-10692-x

**Published:** 2023-03-09

**Authors:** Marion Perréard, Romane Florent, Jordane Divoux, Jean-Michel Grellard, Justine Lequesne, Mélanie Briand, Bénédicte Clarisse, Nathalie Rousseau, Esther Lebreton, Brice Dubois, Valentin Harter, Audrey Lasne-Cardon, Julien Drouet, Alisson Johnson, Anne-Laure Le Page, Céline Bazille, Corinne Jeanne, Martin Figeac, Nicolas Goardon, Dominique Vaur, Emmanuel Micault, Maxime Humbert, Juliette Thariat, Emmanuel Babin, Laurent Poulain, Louis-Bastien Weiswald, Vianney Bastit

**Affiliations:** 1 Normandy University, UNICAEN, INSERM U1086 ANTICIPE (Interdisciplinary Research Unit for Cancers Prevention and Treatment), BioTICLA laboratory (Precision medicine for ovarian cancers), Comprehensive Cancer Center François Baclesse, 3 Avenue du Général Harris, BP 45026, 14 076 Caen, Cedex 05 France; 2 UNICANCER, Comprehensive Cancer Center François Baclesse, Caen, France; 3 Department of Head and Neck Surgery, Caen University Hospital, Caen, France; 4 Normandy University, UNICAEN, SF Normandy Oncology, US PLATON, ORGAPRED core facility, Caen, France; 5 Clinical Research Department, UNICANCER, Comprehensive Cancer Center François Baclesse, Caen, France; 6 UNICANCER, Comprehensive Cancer Center François Baclesse, Biological Resource Center ‘OvaRessources’, Caen, France; 7 IRCBN Institut Régional du cancer Basse Normandie, Biological Resource Center ‘Tumorotheque de Caen Basse-Normandie’, Caen, France; 8 Clinical Research Department, Caen University Hospital, Caen, France; 9 UNICANCER, Comprehensive Cancer Center François Baclesse, North-West Canceropole Data Center, Caen, France; 10 Department of Head and Neck Surgery, UNICANCER, Comprehensive Cancer Center François Baclesse, 3 avenue du Général Harris, Caen, 14000 France; 11 Department of Oral and Maxillofacial Surgery, UNICANCER, Comprehensive Cancer Center François Baclesse, Caen, France; 12 Department of Medical Oncology Surgery, UNICANCER, Comprehensive Cancer Center François Baclesse, Caen, France; 13 Department of Pathology, Caen University Hospital, Caen, France; 14 Department of Biopathology, UNICANCER, Comprehensive Cancer Center François Baclesse, Caen, France; 15 University of Lille, CNRS, Inserm, CHU Lille, Institut Pasteur de Lille, US 41 - UAR 2014 – PLBS, Lille, France; 16 Department of Cancer Biology and Genetics, UNICANCER, Comprehensive Cancer Center François Baclesse, Caen, France; 17 LPC Caen ENSICAEN/CNRS UMR6534, Caen, France

**Keywords:** Head and neck cancer, Patient-derived tumor organoids, PARP inhibitors, Predictive functional assays

## Abstract

**Background:**

Radiotherapy is one of the cornerstones of the treatment of Head and Neck Squamous Cell Carcinomas *(*HNSCC). However, radioresistance is associated with a high risk of recurrence. To propose strategies (such as combinations with drugs) that could over intrinsic radioresistance, it is crucial to predict the response to treatment. Patient-Derived Tumor Organoids (PDTO) are in vitro tridimensional microtumors obtained from patient’ own cancer samples. They have been shown to serve as reliable surrogates of the tumor response in patients.

**Methods:**

The ORGAVADS study is a multicenter observational trial conducted to investigate the feasibility of generating and testing PDTO derived from HNSCC for the evaluation of sensitivity to treatments. PDTO are obtained after dissociation of resected tumors remaining from tissues necessary for the diagnosis. Embedding of tumor cells is then performed in extracellular matrix and culture in medium supplemented with growth factors and inhibitors. Histological and immunohistochemical characterizations are performed to validate the resemblance between PDTO and their original tumor. Response of PDTO to chemotherapy, radiotherapy and innovating combinations are assessed, as well as response to immunotherapy using co-cultures of PDTO with autologous immune cells collected from patient blood samples. Transcriptomic and genetic analyses of PDTO allow validation of the models compared to patients’ own tumor and identification of potential predictive biomarkers.

**Discussion:**

This study is designed to develop PDTO models from HNSCC. It will allow comparing the response of PDTO to treatment and the clinical response of the patients from whom they are derived. Our aim is to study the PDTO ability to predict the clinical response to treatment for each patient in view of a personalized medicine as well as to establish a collection of HNSCC models that will be useful for future innovative strategies evaluation.

**Trial registration:**

NCT04261192, registered February 7, 2020, last amendment v4 accepted on June, 2021.

## Background

### Head and neck squamous cell carcinoma (HNSCC) and treatment modalities

HNSCC is the sixth most common cancer worldwide, with 890,000 new cases and 450,000 deaths in 2018 [1]. It is estimated that locoregional control can be achieved in half of the cases, in part because 65% of patients are diagnosed at advanced stages (T3T4) and also because locoregional relapse is frequent. The prognosis of HNSCC is poor with a 5-year survival rate for all stages around 55% [1].

Treatment of HNSCC is based on several modalities. Surgery is chosen when a total excision is possible. Radiotherapy may be proposed as primary treatment or as adjuvant treatment depending on the existence of poor prognostic factors (margin status, capsular rupture, advanced tumors, etc.) [2]. Systemic treatments such as chemotherapy (cisplatin) or targeted therapy (cetuximab) can be used in combination with radiotherapy. At the recurrent or metastatic phase, HNSCC are often treated with systemic treatments. Immunotherapy has also entered the therapeutic arsenal for HNSCC with the use of anti-PD1 (programmed death-1) agents Nivolumab or Pembrolizumab [1].

The prognosis of HNSCC is burdened by radioresistance, which is partly due to hypoxia and proliferation among other reasons. This explains the lack of effectiveness of radiotherapy in many cases. Research is thus focused on mechanisms factors of radioresistance. Among them, genomic instability of DNA repair systems plays an important role [3]. Molecules affecting DNA repair systems are currently being studied such as PARP inhibitors like Olaparib which was FDA-approved in 2014 since they could allow sensitization of tumor cells to radiotherapy [4]. Another promising field of research is the use of different particles such as proton beam therapy. It has two major advantages over X-rays: more precise energy delivery to the tumor and fewer side effects on surrounding healthy tissues [5].

Patients with HNSCC suffer from numerous complications of treatment. The side effects are increased by therapeutic combinations. These treatments can alter breathing, phonation and swallowing because of the tissue changes caused by the radiation in the areas surrounding the tumor. Patients’ quality of life is altered by functional disorders and by the social impact of treatments. Predicting tumor response and tissue effects from innovative combinations has the potential to improve the therapeutic index.

### Toward predictive medicine

Precision medicine is an innovative approach to tailoring disease treatment that takes into account differences in genetics, environment, and lifestyle of people. The goal of precision medicine is to target treatments to patients who are most likely to benefit from them. Due to the specific problems of HNSCC (radioresistance, high risk of recurrence and secondary cancer, toxicity of treatments), it appears interesting to be able to predict response to conventional treatments using models mimicking the original patient’s tumor. Such models could also be used to test new therapy combinations or new compounds in order to predict their clinical effectiveness and/or the identification of predictive molecular signatures.

Functional assays in precision oncology are based on direct exposure of cancer tissues derived from affected individuals to drugs to predict clinical response. These functional approaches allow to evaluate the response to treatments by considering all the intrinsic characteristics of the tumor without prior molecular analysis. The results can then be correlated to different biological parameters (DNA, mRNA, non-coding RNA, proteins, etc. from various origins like tumor or blood for example) in order to identify predictive biomarkers of treatment response. Functional assays can be performed using various cancer models [6], including Patient-Derived Tumor Organoids (PDTO) which represent an emerging pre-clinical model at the interface of cell lines and patient-derived xenografts mouse models.

PDTO are obtained after dissociation of tumor samples and embedding of tumor cells in extracellular matrix. They are cultured in medium containing a cocktail of growth factors and signaling pathway inhibitors. These culture conditions have been successfully used to establish long-term PDTO lines from many types of cancers [7]. The feasibility of generating HNSCC organoid lines has previously been shown since PDTO from squamous cell carcinoma of the oral cavity [8] and oropharynx [9] have been established in the last few years. These PDTO have the advantages of being able to be amplified relatively quickly after tumor resection, having unlimited potential for proliferation, a high rate of successful establishment, and the ability to be transfected and cryopreserved [10]. These characteristics allow them to recapitulate the cancer spectrum [11]. Moreover, they are close to the original tumor in terms of histological and molecular features [11, 12].

### PDTO could predict the response of the tumor

More and more evidence indicate that PDTO may predict the response of the tumor from which they are derived [7]. A pilot study showed that PDTO from metastatic gastrointestinal tumors predicted the response of 21 patients to different chemotherapies (100% sensitivity, 93% specificity, 88% positive predictive value and 100% negative predictive value) [13]. Another study using PDTO from 11 chemo-naive pancreatic tumors gave a 91% predictive response rate to first-line chemotherapy and 80% to second line chemotherapy [14].

The predictive value of tumor PDTO is also observed for radiotherapy. In their study analyzing 19 patients suffering from colorectal cancer, Park et al. were able to develop a model to predict radio-sensitivity (82% accuracy) and radioresistance (93% accuracy) of patients [15]. Moreover, Driehuis et al. showed that PDTO derived from patients with the worst progression-free survival for HNSCC were more likely to be resistant to radiotherapy [8]. Finally, the response to a combination of chemotherapy and radiotherapy could be efficiently predicted (84% accuracy) using PDTO from colorectal cancer [16]. Altogether, these data highlight the predictive value of PDTO and the relevance to use them to anticipate the response to radiotherapy and its combinations with other treatments. Since the tumor microenvironment also play a critical role in dictating the response to therapies, more and more work is done to complexify the culture of PDTO in order to faithfully recapitulate it. Several trials to culture PDTO with fibroblasts, macrophages or T cells have thus been conducted [17] and gave rise to complex in vitro models. Some of them were used to evaluate the response to immunotherapy in PDTOs derived from melanoma [18] or appendicular cancers [19] and displayed a promising correlation with the clinical response (85 and 100% accuracy respectively). More evidence is still needed to conclude and several trials are currently running to evaluate the predictive value of PDTO in a clinical context [6].

If the predictive value of PDTO model is validated, this would allow testing conventional treatments before administering them to the patient from whom the PDTO are derived. This perspective is very interesting, particularly in HNSCC where more than two thirds of the operated patients will undergo adjuvant treatments such as radiotherapy and/or chemotherapy whose side effects can be important. Despite these treatments, up to half of patients will relapse, highlighting the radio-chemosensitivity heterogeneity of tumors [20]. A predictive screening using PDTO could thus help to refine the choice of treatments adapted to each patient and thus limit adverse effects. As immunotherapies are increasingly considered to treat HNSCC, the development of PDTO models capable to i) assess the efficacy of such treatment and ii) predict patients’ response to these new agents; will be critical in the next few years. In this regard, our study will allow a first set of functional assays in PDTO co-cultured with autologous immune cells and will give initial pieces of evidence about the efficacy of immunotherapies in HNSCC.

## Methods/design

**Fig. 1:**
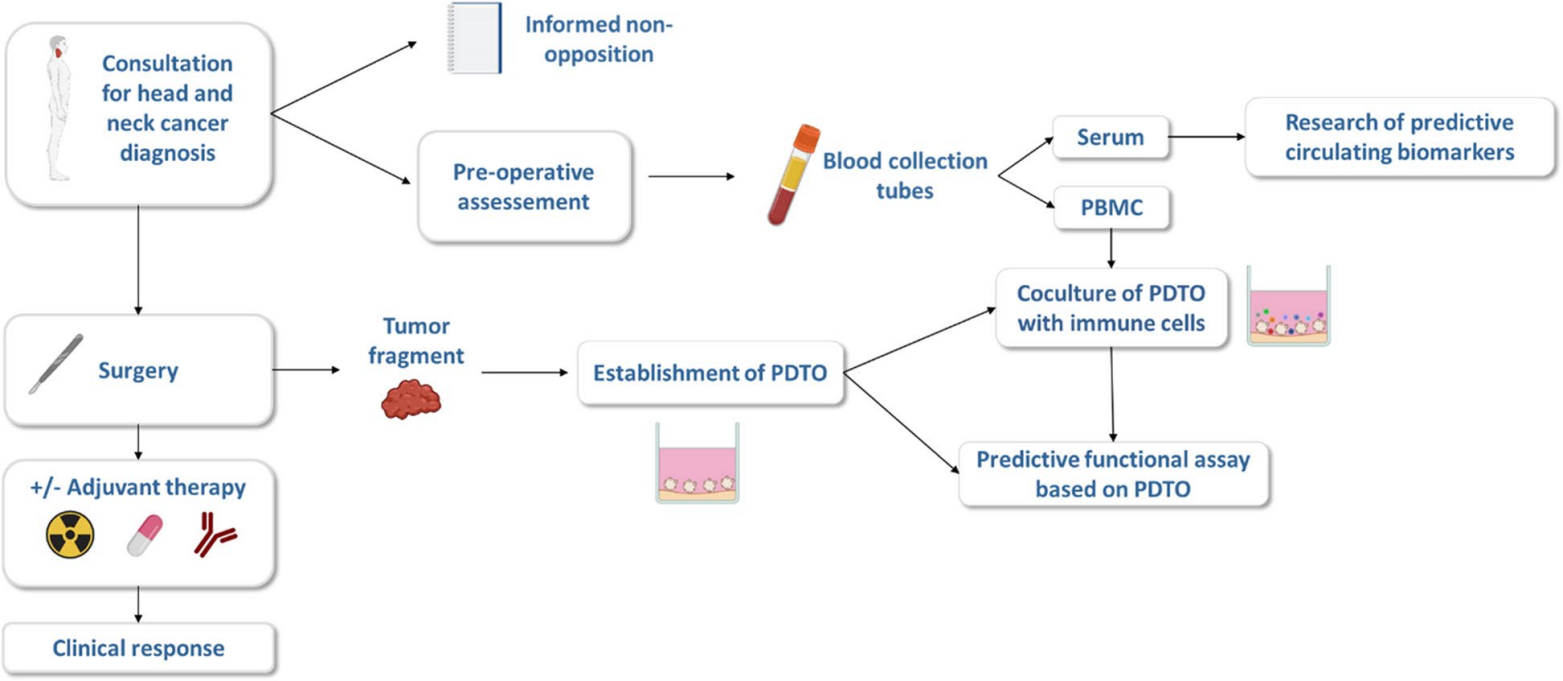
ORGAVADS study design

The ORGAVADS study is a multicenter observational study conducted to investigate the feasibility of generating and testing PDTO from HNSCC for evaluation of sensitivity to treatments (Fig. 1). The ORGAVADS study and this manuscript have been written in accordance with standard protocol items, namely recommendations for interventional trials (SPIRIT).

### Study objectives

The main objective of the study is to assess the feasibility of using PDTO from HNSCC as tools for predicting response to treatments.

The secondary objectives are: i) to assess the feasibility of in vitro functional assays for evaluation of sensitivity to treatments (chemotherapy, radiotherapy, PARP inhibitors and immunotherapy); ii) to investigate the predictive value of PDTO for conventional chemotherapy and radiotherapy; iii) to evaluate the PARP inhibitor-mediated sensitization of radiotherapy; iv) to assess the ability of PDTO to repair DNA lesions by homologous recombination [(HRD (Homologous Recombination Deficiency)/HRI (Homologous Recombination Intermediate)/HRP (Homologous Recombination Proficiency) status); v) to identify molecular signatures for prediction of response (of PDTO and patients) to treatments.

### Study population

Eligibility criteria are described in Table 1. The ORGAVADS study focuses on patients with surgically resectable HNSCC who undergo surgery at François Baclesse Center and Caen University Hospital Center. After patient screening according to criteria, and the patient’s non-opposition, the patient will be enrolled in the study. An identification number will be thus assigned to each patient to be used throughout the study.

**Table 1:** ORGAVADS study inclusion and exclusion criteria

Inclusion criteria	Non-inclusion criteria
Patient ≥ 18 years	Pregnant women
Histologically confirmed squamous cell carcinoma of the oral cavity, oropharynx, hypopharynx or larynx	Patient deprived of liberty or placed under the authority of a tutor
Patients for whom oncologic surgery is planned or who have recently undergone surgery of the tumor of oral cavity, oropharynx, hypopharynx or larynx	
Subject affiliated to a social security regimen	
No opposition to participate to the study	

### Study assessment

The study was approved by the “East III” ethical committee (IDRCB: 2019-A03332-55). Clinicians will inform all patients enrolled in the study that their biological samples could be used for research purposes and patients will express their non-opposition. All donors participating may object at any time, leading to the prompt disposal of their tissue and any derived material, as well as the cessation of data collection. The enrollment period of the study will be 3 years, each patient will be monitored for 5 years and study length will be 10 years.

#### Collection of tumor and blood samples

Blood samples will be collected before surgery as part of regular medical care. No blood draw will be done specifically for this study. An additional volume of blood will be collected in two 5 ml serum separator tubes and seven 5 ml EDTA tubes and transported to the laboratories to be processed.

Tumor samples will be collected during surgery as part of regular medical care and sent to the pathology laboratory. The surplus tissue (including tumor and adjacent normal tissues) not required for diagnosis will be transported to the laboratory in vials containing medium supplemented in antibiotics and ROCK inhibitor Y-27632.

#### Sample processing

##### Serum preparation

Serum will be isolated by a two-step centrifugation (2500 g, 10 min, RT), and aliquoted (about 5 tubes of 300 μL) before storage at − 80 °C.

##### Isolation of Peripheral Blood Mononuclear Cells (PBMC)

PBMC will be isolated from human peripheral blood by density gradient centrifugation using Ficoll-Paque in Leucosep tubes. Then, cells will be resuspended in cold Fetal Bovine Serum (FBS), and counted. PBMC will be resuspended in freezing solution (10% DMSO, 90% non-inactivated FBS), aliquoted (about 5 cryovials, 4.10^6^ cells/cryovial), and frozen with gradually decreasing temperatures (1 °C/min) to − 80 °C before long-term storage at liquid nitrogen temperatures in stored in the Biological Resource Center ‘Tumorotheque de Caen Basse-Normandie’ (NF-S 96900 quality management, AFNOR No. 2016: 72860.5).

##### Tumor sample

Tumor specimen will be cut into 1-3 mm^3^ pieces. Depending on the quantity of tumor, different procedures will be carried out in the following order of priority: 1) one random piece will be processed for the isolation of viable cells and PDTO establishment; 2) two random pieces will be snap frozen and stored at − 80 °C for molecular analyses; 3) One random piece will be fixed in paraformaldehyde for paraffin embedding and subsequent histopathological analysis and immunohistochemistry; 4) the remainder will be cryopreserved in freezing solution (10% DMSO, 90% non-inactivated FBS) for future isolation of viable cells. All tumor samples will be stored in the Biological Resource Center ‘Tumorotheque de Caen Basse-Normandie’.

#### Medical data collection

Medical data collected for each patient in order to correlate biological data (including PDTO response to treatment) with clinical response are indicated in Table 2.

**Table 2:** Medical data collected for each patient

Clinical parameters	Age, sex, weight, height, ECOG performance status, Exposure to tobacco and alcohol, Cancer location and TNM-8 classification
Histological diagnosis	Type, squamous differentiation, keratinization type, p16 status
Biologic parameters	Serum neutrophils, albumin and leukocytes levels
Pathological pronostic factors	Surgical margin, lymph node metastasis with or without capsular rupture, vascular or lymphatic embolization, perineural invasion
Oncologic treatments	Radiotherapy (volume, dose, fraction) Chemotherapy (molecules, administration regimen)
Response to treatment	Disease-free survival, Progression-free survival, Overall survival.

#### Establishment of panel of PDTO

**Fig. 2:**
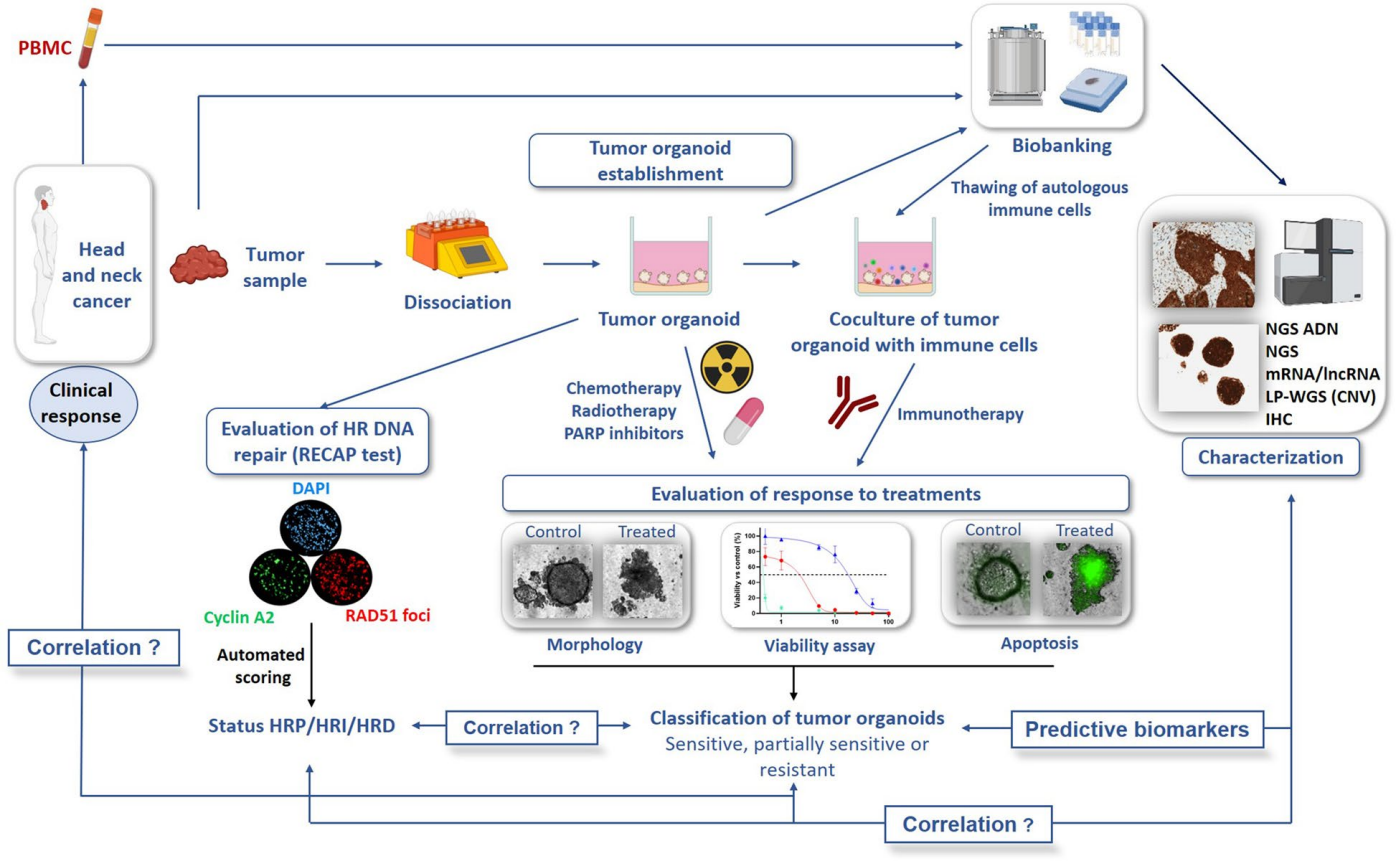
Establishment and characterization of PDTO derived from HNSCC and evaluation of response to treatments to assess its predictive value

Tumor pieces will be dissociated enzymatically and mechanically to obtain isolated cells or small cell clusters (Fig. 2). Cells will be embedded in an extracellular matrix (growth factor-reduced Matrigel or BME II) and cultured in a medium supplemented with growth factors and signal pathway inhibitors [Advanced DMEM (Gibco) supplemented with 100 UI/mL of penicillin and streptomycin (Gibco), 1% GlutaMAX (Gibco), 1X B27 (Gibco), 1.25 mM NAC (Sigma-Aldrich), 50 ng/mL EGF (PeproTech), 10 ng/mL FGF-10 (PeproTech), 5 ng/mL FGF-b (PeproTech), 500 nM A-83-01 (PeproTech), 10 μM Y27632 (Interchim), 10 mM Nicotinamide (Sigma-Aldrich), 1 μM PGE2 (PeproTech), 1 μM Forskolin (Peprotech), 0.3 μM CHIR99021 (Biogems), 100 μg/mL Primocin (InvivoGen), 50% Wnt3a, RSPO3, Noggin-conditioned media (L-WRN, ATCC), and 10% RSPO1-conditioned media (Cultrex HA-R-Spondin-1-Fc 293 T, Amsbio)]. Culture medium will be changed twice a week. Once formed, PDTO will be dissociated and reseeded to amplify them for experimental purposes. Cryovials will be prepared at regular intervals by dissociating and resuspending PDTO in Recovery Cell Culture Freezing Medium (Gibco) prior to be biobanked in liquid nitrogen. It should be noted that PDTO line will be considered as established when it will be maintained for more than 3 passages. For each PDTO line, samples will be kept frozen for DNA/RNA/protein analysis and others will be embedded in paraffin for histopathological analysis. This will allow comparisons between the characteristics of the PDTO and the tumor from which they are derived in order to validate their correspondence.

#### Coculture of PDTO with immune cells

Co-culture of PDTO and autologous immune cells will be based on the protocol described by Cattaneo et al. [21] Briefly, PDTO specific T cells will be induced through serial co-culture with dissociated PDTO. PBMC (~ 10.10^6^ cells) will be thawed and set to resting condition with IL-2 (150 U/mL) overnight. Meanwhile, PDTO will be treated with IFNγ (200 ng/mL) for 24 hours to favor antigen presentation and will be dissociated to produce Antigen Presenting Tumor Cells (~ 0.5.10^6^ cells APTC). PBMC and APTC will be next co-cultured (20:1 ratio) in a CD28 (5 μg/mL) coated culture plate for successive periods of 7 days to induce clonal expansion of PDTO specific T cells. T cells will be then evaluated for their tumor reactivity through the detection of activation (CD137) and functional (CD107a, IFNγ) markers by flow cytometry (Cytoflex, Beckman Coulter) and will be cryopreserved for later use for the evaluation or response to treatments.

#### Evaluation of response to treatments

The evaluation of the response to treatments will be performed when PDTO have reached a diameter of 150 μm in « PDTO treatment medium », corresponding to the PDTO culture medium lacking N-Acetylcysteine, Y-27632 and primocin.

PDTO will be collected, resuspended in 2% extracellular matrix/PDTO culture medium and then platted in white and clear bottom 96-well plates previously coated with a 1:1 volume mix of PDTO treatment medium with extracellular matrix. In the case of evaluation of the response to radiotherapy, PDTO will be before irradiated using the CellRad System (FAXITRON Bioptics). In the case of evaluation of the response to chemotherapy or PARP inhibitors, drugs are prepared in 2% extracellular matrix/PDTO culture medium and added 1 hour after PDTO have been plated.

In the case of evaluation of the response to immunotherapies, PDTO will be co-cultured with PDTO specific T cells previously generated (*see co-culture of PDTO with immune cells*) at a 5:1 ratio. Treatments (such as Nivolumab or Pembrolizumab) will be added directly in the co-culture. A condition containing an MHC-I blocking antibody will be added to control for antigen specific killing.

PDTO morphology will be monitored by taking images during the required time using Incucyte S3 (Sartorius). At the endpoint, PDTO response will be assessed using CellTiter-Glo 3D cell viability assay (Promega) according to the manufacturer’s instruction and luminescence will be measured using GloMax Discover GM3000 (Promega) with the associated software. Results will be normalized to the control condition. IC50 will be calculated with GraphPad software. The ability of T cells to recognize and induce lysis of PDTO will be monitored via analysis of caspase 3 cleavage within PDTO and visualization of LAMP-1 on the membrane of CD8+ T cells.

The treatment response of the PDTO will be finally compared to the clinical response (PFS/DFS/OS) of the patient from whom they are derived in order to validate the predictive value of this model for HNSCC.

#### Evaluation of PDTO model relevance and identification of potential predictive biomarkers

##### Transcriptomic analysis

Total RNA will be extracted using the NucleoSpin miRNA kit (Macherey Nagel, Hoerdt). Libraries will be made by the Genomics Platform of the University of Lille with the QuantSeq 3’RNA Library Kitto allow polyA mRNA and lncRNA recovery. We will add ERCC spike-in for quality control. Library generation is initiated by oligo dT priming, from total RNA (200 ng). The primer already contains Illumina compatible linker sequences (Read 2). After first strand synthesis the RNA is degraded and second strand synthesis is initiated by random priming and a DNA polymerase. The random primer also contains 5′ Illumina compatible linker sequences (Read 1). At this step Unique Molecular Identifiers (UMIs) are introduced allowing the elimination of PCR duplicates during the analysis. After obtaining the double stranded cDNA library, the library is purified with magnetics beads and amplified. During the library amplification the barcodes and sequences required for cluster generation (index i7 in 3′ and index i5 in 5′) are introduced due to Illumina compatible linker sequences. Library is amplified with 14 PCR cycles. The final library is purified and deposed on High sensitivity DNA chip to be controlled on Agilent bioanalyzer 2100. The library concentration and the size distibution is checked. Each library is then pooled equimolarly and the final pool is also controlled on Agilent bioanalyzer 2100 and sequenced on NovaSeq 6000 (Illumina) with 100 cycles chemistry aiming to obtain a minimum of 20 M reads by sample.

To eliminate poor quality regions and poly(A) of the reads, we use the program fastp with quality score threshold of 20. We also remove reads shorter than 25 pb. The read alignments are performed using the program STAR with the genome reference human (GRCh38) and the reference gene annotations (Ensembl). The UMI (Unique Molecular Index) allowed to reduce errors and quantitative PCR bias are processed using fastp and umi-tools. Based on reads alignements, we counted the numbers of molecules by gene using FeatureCount. Others programs are performed for quality control as qualimap, fastp, FastQC and MultiQC. Statistical analysis is realized with the R/bioconductor package DESeq2.

##### Copy number variations analysis by low-pass whole genome sequencing

WGS will be performed using Illumina DNA PCR Free prep kit, starting with 500 ng of DNA. Library will be sequenced on a NovaSeq 6000 system aiming at 6x depth. Copy Number Analysis will be performed with HMMcopy and ichorCNA.

##### Transcriptome and CNV analysis

A preliminary analysis of intra reproducibility and differences between original tumors and PDTO assessed by PCA and unsupervised hierarchical clustering for each omic data after normalization will be done. For each sample we will plot barplot based on the normalized count of reads or number of aberration (CGH) for specific pathways to evaluate heterogeneity in each type of samples. Studied pathways will be defined with ingenuity pathways analysis (proliferation, apoptosis, etc). Each PDTO will be compared to original tumors data.

##### Targeted sequencing by NGS

“Mutational status (SNV, indels, CNV and translocations)” will be established on original tumor samples and PDTO derived from the primary tumor by targeted re-sequencing of a panel of 580 genes involved in cancer for DNA and of a panel of 57 genes implicated in protein fusion for RNA. DNA and RNA from samples will be extracted using QIAamp DNA kit (Qiagen) and NucleoSpin miRNA kit (Macherey Nagel, Hoerdt) respectively. DNA and RNA libraries sample preps and targeted enrichment will be performed using SureSelect XT HS2 DNA Reagent Kit (Agilent) and SureSelect XT HS2 RNA Reagent Kit (Agilent) respectively according to manufacturer’s instructions. Paired-end sequencing will be realized on a Nextseq500 sequencer from Illumina with a mean depth of coverage of 1000X. Bioinformatic analysis will be performed using an already developed pipeline. Briefly, duplicate reads merge, using unique molecular identifier, will be based on AGeNT (Agilent). The alignments will be based on BWA and GATK for BAM recalibration. Variant calling will be performed using Haplotype caller, Lofreq and OutLyzer. Finally, variant annotation will be realized with Alamut Batch and Annovar. The evaluation of copy number of targeted genes (Gain or Loss) will be evaluated using already published softwares such as ONCOCNV, CNVkit or current relevant tools.

### Statistical consideration

#### Sample size determination

To estimate the successful PDTO establishment rate, assumed around 60%, with a 95% confidence interval of 20% width, 89 tumor samples will be required. Anticipating 10% of non-assessable samples, it is planned to include 98 patients.

#### Statistical analyses

Qualitative variables will be described using the sample numbers and percentages. Quantitative variables will be described using the mean (+/− standard deviation) or the median and the range if normality hypothesis is not verified*.*

To address the primary objective, the rate of successful PDTO establishment, i.e., the rate of tumor samples usable for predictive functional assays based on PDTO, will be estimated with its 95% confidence interval. Then, PDTO response to treatment will be correlated with the clinical response by performing a Chi2 test and computing the Cohen’s kappa coefficient. Associations between biological parameters and clinical response will be assessed by one-way analysis of variance (or the non-parametric Kruskal-Wallis test, if necessary). Receiver Operating Characteristic (ROC) curves and a logistic regression model will also be used to identify predictive factors of clinical response.

An alpha level of 5% will be considered to indicate statistical significance for each statistical analysis and confidence interval.

### Data management

Medical data management will be performed by an independent data management center. The data collection will be done using an eCRF (electronic Case Report Form) and in accordance with the study protocol. After checking accuracy and completeness of the recorded data, the eCRFs will be signed by the investigator and archived by the data management center.

## Discussion

PDTO are preclinical models that mimic morphological and genetic characteristics of their original tumors [8]. Recent studies on various tumor types such as colorectal and urogenital cancers appear to show a correlation between the response of PDTO to treatment and the clinical response of patients [7]*.* These results are promising but not well studied in HNSCC.

We propose to perform this clinical study to compare the response to treatment of PDTO in HNSCC and the clinical response of the patients from whom they are derived. Our aim is both to demonstrate the interest of PDTO as predictive tools for each patient in view of a personalized medicine, and to use the established HNSCC tumor organoids collection for preclinical evaluation of new therapeutic compounds/strategies as well as for the identification of predictive molecular signatures. Eventually, PDTO could allow a better selection of treatments to which the tumors would be most sensitive and to limit the sequelae related to potentially less effective treatments, thus finding their place in true precision oncology protocols.

## Data Availability

Not applicable.

## Abbreviations

APTC: Antigen Presenting Tumor Cells
CNV: Copy Number Variation
CGH: Comparative Genomic Hybridization
DFS: Disease Free Survival
eCRF: electronic Case Report Form
FBS: Fetal Bovine Serum
ENT: Ear Nose and Throat
FDA: Food and Drug Administration
HNSCC: Head and Neck Squamous Cell Carcinomas
HR: Homologous Recombination Deficiency
HRI: Homologous Recombination Intermediate
HRP: Homologous Recombination Proficiency
NGS: Next Generation Sequencing
OS: Overall Survival
PARP: poly-ADP-ribose-polymerase
PBMC: Peripheral Blood Mononuclear Cells
PCR: Polymerase Chain Reaction
PD1: Programmed death-1
PDTO: Patient-Derived Tumor Organoids
PFS: Progression Free Survival
ROC: Receiver Operating Characteristic
UMIs: Unique Molecular Identifiers
